# Functional variability of two lignification genes in *Eucalyptus urophylla*

**DOI:** 10.1186/1753-6561-5-S7-O12

**Published:** 2011-09-13

**Authors:** Eric Mandrou, Frédéric Mortier, Marie Denis, Gilles Chaix, Emilie Villar, Christophe Plomion, Jean-Marc Gion

**Affiliations:** 1 INRA-CIRAD, France; 2 CIRAD, France; 3 CIRAD-INRA, France; 4 INRA, France

## 

Lignin quantity and composition are major components of wood quality in eucalypts breeding programs. Optimizing these traits can have a huge economical impact for charcoal and pulp production. However, little is known about the genetic determinism of these traits. Our objective is to establish efficient early selection criteria to identify ideotypes for these traits using gene based markers. To reach this goal, we first estimated genetic parameters for lignin quantity and composition using a factorial design comprising 16 founders and 328 progenies of *E. urophylla*. We found high heritability for both Klason Lignin (hÂ²=0.85) and Syringyl to Guaiacyl ratio (S/G: hÂ²=0.62). Then, nucleotide and haplotype diversities were described for two linked genes involved in the lignification process (*CCR* and *ROP1*). High levels of nucleotide diversity and rapid decay of linkage disequilibrium were observed in a sample of 16 trees. A SNP by SNP association mapping was carried out in the 328 progenies using a mixed linear model (available in Tassel). A total of 4 SNP (3 in *CCR* and one in *ROP1*) were significantly associated with S/G ratio explaining between 1 and 1.8% of the trait variation. Finally, the additive effects of haplotypes (defined by all SNPs) were estimated using a Bayesian approach of the mixed model including the pedigree (MCMCglmm package available in R software). This analysis confirmed the existence of two distinct additive effects for *CCR* and *ROP1* on S/G ratio, suggesting the existence of two S/G QTLs for these two linked genes in *E. urophylla*.

